# Barriers to and Facilitators of a Blended Cognitive Behavioral Therapy Program for Depression and Anxiety Based on Experiences of University Students: Qualitative Interview Study

**DOI:** 10.2196/45970

**Published:** 2023-04-12

**Authors:** Pia Braun, Ece Atik, Lisa Guthardt, Jennifer Apolinário-Hagen, Magnus Schückes

**Affiliations:** 1 Institute of Occupational, Social and Environmental Medicine, Faculty of Medicine, Centre for Health and Society Heinrich Heine University Düsseldorf Düsseldorf Germany; 2 Translational Psychotherapy, Georg-Elias-Müller-Institute of Psychology University of Göttingen Göttingen Germany; 3 Institute for SME Research and Entrepreneurship University of Mannheim Mannheim Germany

**Keywords:** digital therapeutics, blended cognitive behavioral therapy, bCBT, depression, anxiety, acceptance, user experiences, university students, mobile phone

## Abstract

**Background:**

Blended cognitive behavioral therapy (bCBT) programs have been proposed to increase the acceptance and adoption of digital therapeutics (DTx) such as digital health apps. These programs allow for more personalized care by combining regular face-to-face therapy sessions with DTx. However, facilitators of and barriers to the use of DTx in bCBT programs have rarely been examined among students, who are particularly at risk for developing symptoms of depression and anxiety disorders.

**Objective:**

This study aimed to evaluate the facilitators of and barriers to the use of a bCBT program with the *elona therapy* app among university students with mild to moderate depression or anxiety symptoms.

**Methods:**

Semistructured interviews were conducted via videoconference between January 2022 and April 2022 with 102 students (mean age 23.93, SD 3.63 years; 89/102, 87.2% female) from universities in North Rhine-Westphalia, Germany, after they had completed weekly individual cognitive behavioral therapy sessions (25 minutes each) via videoconference for 6 weeks and regularly used the depression (n=67, 65.7%) or anxiety (n=35, 34.3%) module of the app. The interviews were coded based on grounded theory.

**Results:**

Many participants highlighted the intuitive handling of the app and indicated that they perceived it as a supportive tool between face-to-face sessions. Participants listed other benefits, such as increased self-reflection and disorder-specific knowledge as well as the transfer of the content of therapy sessions into their daily lives. Some stated that they would have benefited from more personalized and interactive tasks. In general, participants mentioned the time requirement, increased use of the smartphone, and the feeling of being left alone with potentially arising emotions while working on tasks for the next therapy session as possible barriers to the use of the app. Data security was not considered a major concern.

**Conclusions:**

Students mostly had positive attitudes toward *elona therapy* as part of the bCBT program. Our study shows that DTx complementing face-to-face therapy sessions can be perceived as a helpful tool for university students with mild to moderate anxiety or depression symptoms in their daily lives. Future research could elaborate on whether bCBT programs might also be suitable for students with more severe symptoms of mental disorders. In addition, the methods by which such bCBT programs could be incorporated into the university context to reach students in need of psychological support should be explored.

## Introduction

### Background

Depression and anxiety disorders remain among the leading causes of burden worldwide and have severe consequences for those affected, impairing their mental and physical health as well as their social lives [1-3]. University students are particularly at risk for developing symptoms of depression and anxiety disorders. A systematic review by Paula et al [4] reported a prevalence of 24.5% for anxiety symptoms and 26.1% for depressive symptoms among university students. According to Kessler et al [5], two-thirds of the related symptoms emerge before the age of 25 years. Major issues that affect students’ well-being and academic performance seem to be psychological instability that is due to a substantial life transition, stress, and financial uncertainty [6-8], leading to a significant increase in the demand for counseling services and therapy, such as cognitive behavioral therapy (CBT) [9]. CBT has consistently been shown to be effective in treating depression and anxiety disorders and has become the gold standard for psychotherapy in this field [10]. Barriers to the large dissemination of CBT include long waiting lists [11], stigma associated with seeking help [12,13], and low flexibility owing to difficulties in scheduling and attending therapy sessions [14].

Internet-based CBT (iCBT) is widely acknowledged as a useful and effective resource for increasing access to mental health care [15,16], especially for digital natives such as students [17]. iCBT programs comprise an electronic, standardized treatment program either with (ie, guided iCBT) or without (ie, unguided iCBT) therapeutic support via chat, email, or calls [18]. In an unguided format, the effectiveness of iCBT, including digital health apps, seems to be limited [19,20], and dropout rates appear to be greater when the intervention does not involve therapist contact [21,22]. Both guided and unguided iCBT programs normally follow a standardized course content protocol that does not leave much room for individualization according to personal needs [14], and this could be associated with patients’ relatively low willingness to use iCBT programs compared with face-to-face interventions [23,24].

An evolution of iCBT is a blended CBT (bCBT) program that integrates regular face-to-face CBT sessions with digital therapeutics (DTx) such as evidence-based mental health apps to mitigate the disadvantages of iCBT while benefiting from several advantages [25,26]. The bCBT appears to be an acceptable, clinically effective, and cost-effective option for treating depression and anxiety disorders [27-32]. Given that the therapy is augmented by DTx, it has the potential to decrease the number of face-to-face sessions with therapists and increase their resources to treat more patients. In addition, research has shown that bCBT achieves similar outcomes to traditional CBT, despite reduced face-to-face time with therapists [27,33-35]. As the therapist is in charge of the therapy in bCBT, more personalized care is possible compared with iCBT, that is, by selecting modules and exercises within DTx that are most relevant to the client’s needs and goals, which might enhance motivation and compliance [36]. Tailored interventions also seem to be a prerequisite to increasing university students’ intention to use mental health services because *one-size-fits-all* approaches are unlikely to be effective for everyone [37,38]. Thus, to increase treatment uptake among students with depression or anxiety symptoms, there is a need for bCBT approaches that fit therapists’ as well as patients’ needs and preferences [39]. A few studies have focused on therapists’ perspectives regarding their expectations for and experiences with bCBT [38,40], but to date, there is little qualitative research on patients in general and with students in particular. A study by Etzelmueller et al [41] reported predominantly positive experiences with a bCBT program among patients with major depressive disorder. For the same target group, Urech et al [42] could identify different perceived advantages and disadvantages of bCBT after undergoing such a program for 18 weeks. However, for students as a promising target group, research on their experiences with bCBT is still scarce.

### Objective

In light of these developments, we investigated experiences with a bCBT program accompanied by a novel digital health app (*elona therapy*) that offered an integrated synthesis of digital and face-to-face elements, with individualization promoted by allowing therapists to regularly adapt relevant therapeutic content that fits the symptomatology and personal needs of patients. The content could be accessed by patients’ smartphones. The aim of this study was to evaluate the benefits of the app for use in bCBT through the eyes of student users with mild to moderate depression or anxiety as well as factors that might be associated with its use (and nonuse) in this sample. To date, even though young adults seem to be particularly suitable for bCBT, facilitators of and barriers to the use of these apps, such as *elona therapy*, have rarely been examined in university students.

## Methods

### Design

This qualitative study was conducted as part of a feasibility and effectiveness study addressing bCBT intervention programs for depression or anxiety in university students [32]. A total of 107 students with mild to moderate depression or anxiety symptoms (ie, Patient Health Questionnaire-9 or Generalized Anxiety Disorder-7 scores between 5 and 15) [43,44] participated in either depression or anxiety intervention programs depending on their symptomatology. Participants with both elevated depression and anxiety symptoms were assigned to one of the intervention groups based on a decision made jointly by the participant and the clinical psychologist conducting the interview (shared decision-making). The bCBT programs included weekly individual CBT sessions (25 minutes each) with a therapist via videoconferencing for 6 weeks. To support weekly therapy sessions, students used either the depression or the anxiety module of *elona therapy* on their smartphones throughout the intervention duration. Weekly CBT sessions with therapists included interactive therapeutic tasks and joint discussions. In addition, the app gave students access to supporting digital exercises and psychoeducational resources, which could be customized by the therapist according to the students’ needs. Therapy sessions and supporting digital homework were developed as a manual separately for students with depression and anxiety. This basis level of intervention, defined by manuals, was given to all participants depending on their symptomatology. In addition, therapists had the option to activate additional digital content (eg, psychoeducational tasks or therapeutic activities) for each student based on their individual needs. The depression module of *elona therapy* provided psychoeducation and techniques and interventions related to behavior, thoughts, emotions, and relationships. It also included a specific module on relapse prevention. The anxiety module of *elona therapy* provided psychoeducation techniques; interventions related to the factors that contribute to its maintenance and how thoughts and emotions are related to anxiety; and specific modules on exposure techniques, acceptance and commitment therapy techniques, and relapse prevention. A more detailed description of the session manual and available content of the app can be found in the study by Atik et al [32]. Students who had completed the intervention program were invited to semistructured individual interviews to elaborate on their experiences with the bCBT program.

### Participants and Recruitment

All students who had completed the bCBT intervention program were invited for a final interview by their therapist. Information on the interviews was provided during the last videoconference therapy session. Participants were informed that the interviews would consist of a discussion on their experiences in the program and that it would be guided by an independent experienced interviewer who was not involved in the therapeutic process. Semistructured interviews were conducted with 102 university students. The female and male interview participants were 87.2% (89/102) and 12.7% (13/102), respectively, and their ages ranged from 19 to 38 years with a mean age of 23.93 (SD 3.63) years. Table 1 displays the sample characteristics. Interview participation was considerably high; 95.3% (102/107) of the participants in the intervention study agreed to participate in the feedback interview.

**Table 1:** Sample characteristics (N=102).

Characteristics		Values
Age (years), mean (SD)		23.93 (3.63)
Female, n (%)		89 (87.2)
**bCBT^a^ program, n (%)**		
	Depression	67 (65.7)
	Anxiety	35 (34.3)
**University major, n (%)**		
	Social sciences and humanities (eg, languages, sociology, education, economics, etc)	39 (38.2)
	Medicine and related fields (eg, dentistry, pharmacy, etc)	18 (17.8)
	Science and engineering	16 (15.7)
	Psychology	14 (13.7)
	Management and business administration	7 (6.9)
	Recent graduates (nonuniversity student)	7 (6.9)
	Sports	1 (1)

^a^bCBT: blended cognitive behavioral therapy.

### Interview Procedure

Interviews were conducted on the web via videoconference between January 2022 and April 2022 and were audio recorded with the consent of the participants. The interviewer (EA) informed the participants that the goal of the study was to explore their experiences with the bCBT program. EA is a graduate psychologist and researcher trained in qualitative methods. There was no relationship between the interviewer and participants before this study. The interviewer was aware that the bCBT group (depression or anxiety) participants had been assigned to in the main study. The interviews lasted for an average of 22 (SD 7) minutes. Interview recordings were stored and transcribed anonymously. Participants did not receive any financial compensation for their participation neither in the intervention study nor in the feedback interviews.

A general interview guide was used to conduct the semistructured interviews (Textbox 1). On the basis of the responses given by the participants to the previous question, more precise interview questions were carefully chosen and addressed to the participants. EA and MS created the interview guide with the help and synthesis of interview guidelines from a number of studies that qualitatively examined user experiences in applications developed in the field of digital health [45-49]. The interview guide was adjusted to examine emerging categories and themes during the interview process.

**Interview guide for semistructured user interviews.**

**Questions**

- What motivated you to participate in the blended cognitive behavioral therapy (bCBT) program?
- What were your experiences and perceptions of bCBT with *elona therapy*? (emotions, cognition, and process)
- Did the bCBT program help you?
  If yes, how?
  If no, why not?
- How difficult or easy was it for you to spend time with *elona therapy* regularly, that is, to integrate it in your daily life?
- What motivated you to use *elona therapy*?
- What discouraged you from using the app?
- Did the use of the bCBT program affect your knowledge about depression (anxiety disorders)? How so?
  If yes: Do you think this increased knowledge made an impact on your symptoms and mood?
- Did the use of the bCBT program bring you any other personal gains or competencies? (as in increased knowledge)
- Did the use of the bCBT program caused any drawbacks?
- How did you perceive the quality of the modules in *elona therapy*?
  What did you like?
  What did you dislike?
- Did you feel comfortable providing personal information to the app?
- Do you think blended therapy offers additional value compared with usual psychotherapy? Why?
- Do you think blended therapy creates additional burden to the usual psychotherapy? Why?
- In your experience, were there any essential preconditions to make bCBT feasible? If yes, what are they?
- Would you recommend the bCBT program you have attended to your friends and family?

### Data Analysis

The transcripts were coded based on grounded theory, a systematic data analysis methodology that focuses on inductively developing abstract theoretical conceptions from empirical data [50,51]. A qualitative study design and the use of grounded theory have been especially well suited to accomplish our research goal because they enable the examination of emergent patterns and themes directly from participant data without assumptions from prior research or theories [52]. For the coding procedure of the transcriptions, Dedoose software (version 8.0.35, 2018; SocioCultural Research Consultants, LLC) for qualitative research was used.

The coding procedure began after the interview process had been concluded. All the interviews were coded individually and chronologically. Multiple researchers conducted the coding procedures to ensure intercoder reliability. The first round of data coding was completed by a student research assistant, with EA reviewing the coding scheme. EA then performed a second round of coding. Third, PB, EA, and MS revisited the coding scheme and discussed some modifications with JAH. Finally, a third round of coding was performed by PB after the authors settled on the final categories. Because cross-checking revealed only minor changes, 3 coding rounds were deemed sufficient. Four aggregate dimensions (use patterns of the app, factors that motivated people to use the bCBT program, benefits of the app, and the facilitators of and barriers to the use of the app) and several subcategories within these aggregate dimensions were formed by inductive category formation based on the content of the interviews and codes assigned to the different text passages.

Interview quotes in this study were translated from German into English by LG. LG has a master’s degree in literary translation and is experienced in translating from German to English. In Multimedia Appendix 1, we report the complete checklist of COREQ (Consolidated Criteria for Reporting Qualitative Research) [53].

### Ethics Approval

Ethics approval for this study was obtained from the Ethical Board of the University of Mannheim (EK Mannheim 27-A/2021) and was part of a joint ethics approval granted to the main study [32].

## Results

### Overview

We discovered general use patterns of the app as well as the barriers to and facilitators for the use of the app in a bCBT program in the analysis of interviews. The hierarchical representation of the categories, themes, and dimensions that emerged from the coding of user interviews are presented in Table 2.

**Table 2:** Hierarchical representation of the categories and dimensions according to the results.

Third order: aggregate dimensions and second order: themes		First order: categories
**Use patterns of the app**		
	Common use patterns	Several times a week Once a week (one day before therapy session)
**Benefits of the app**		
	Self-efficacy	More intensive examination of the therapy content at home Preparing therapy sessions with the help of the app
	Transfer into daily life	Work on mental health whenever and wherever the patient wants Patient takes personal time for their mental health beyond sessions Patient takes more initiative and control in their therapy
	Psychoeducation	More knowledge about emotions and the underlying mental health disorder More self-reflection owing to increased knowledge and awareness on personal problems and their origins Accepting and respecting mental disorder as a disease
**Facilitators of the use of the app**		
	Usability and structure	Good overview (good structure of courses, therapy progress, and onboarding, and good intuitive handling) Comprehensibility of the tasks Praising after task completion Reminder notifications
	Content and design	Appropriate and professionally created modules and videos Nice and modest design
	Support	App as a caring companion throughout the week App provides structure for the students’ lives
**Barriers to the use of the app**		
	Overwhelming emotions	Being left alone with difficult feelings that could arise during reflections and tasks Compulsory tasks may create pressure
	Time requirement	Increased time spent on psychotherapy can be a burden Increased involvement with the smartphone
	Data security concerns	Concerns about data safety and anonymity

### Use Patterns of the App

Overall, students self-reported that they had engaged with the app for 20 to 30 minutes in a given week. The shortest app use estimation reported by a student was 15 minutes, and the longest was 90 minutes per week. Participants also highlighted that no prior knowledge was needed to participate in the bCBT program and to work with the app.

In the program, 2 main patterns emerged regarding students’ preferences for app use. The first pattern was to use the app several times a week by splitting assigned homework tasks over different days. Students who followed this pattern reported spontaneous engagement with the app and stated that they did not plan a time slot for doing the homework and did parts of the homework at will throughout the day. The second pattern was to complete all assigned tasks just before the weekly session with their therapist, usually the day before their session. Students who engaged with the app in this manner thought that they were able to easily remember the content of the homework in sessions this way. Some students also alternated between these 2 patterns:

Sometimes, the exercises were shorter, and then I did all of them at once. With longer ones, I took more time to think about them and to do them in more detail, so I rather split the tasks.

P72

### Benefits of the App

#### Transfer Into Daily Life

Students indicated that they benefited from using the app because they were able to initiate changes in their daily lives. Some highlighted that the structure of the therapy module as well as the relatively short exercises were ideal for daily use and could be easily integrated into stressful periods. The content of the modules was mostly perceived as helpful to apply to themselves, such as the following exercise: participants had to think of certain deconstructive thoughts in an everyday situation and then of alternative, more realistic thoughts. These exercises were evaluated as helpful add-ons to therapy sessions because they could be performed alone at home. Students also imagined that what they had learned throughout the bCBT would be reinforced with a little practice over time:

I think, if you keep using it, you recognize certain patterns in your daily life. Once you have more experience, you might notice, alright, this could be a cognitive bias, let’s try a different perspective. Or that you do something nice for yourself even though you’re not feeling quite well. I think the exercise could also help improve that.

P72

In addition, as the app was used in patients’ own time, the more intensive examination of the therapy content was possible, and this was perceived as supportive in the preparation for the next therapy session. Some of the interviewed students highlighted that they could think about specific themes that were raised during the therapy session in more detail and that they could receive more information about their symptoms, which was considered to positively influence the therapeutic outcome:

The blended way is definitely better, especially for taking initiative even before the actual session with the therapist, just to gather your thoughts and to get some information beforehand.

P17

However, some of the interviewed students wished for even more personalized care, such as daily exercises specific to their current situation and symptoms because it might more effectively help them to combat depressive or anxiety episodes in their everyday lives:

Some more specialized help would be nice, maybe also some daily exercises beyond the app. So that you calm down a bit or recollect things, something like that. Some mental exercises to instantly combat bouts of depression. And it would be great to have it personalized, tailored at different forms of depression and states of anxiety.

P83

A couple of participants mentioned that the app would profit from more interactive tasks because exercises that actively involved students were often seen as particularly helpful:

It might be nice to increase the number of interactive tasks in which you have to enter something yourself. And to have even more possibilities to interact with the app yourself...But what I liked best were the exercises where you had to become active and do something yourself.

P74

#### Self-efficacy

Furthermore, the interviewed students highlighted the flexibility of complementing the CBT with the app. Some participants pointed out that they could work on their mental health whenever and wherever they wanted, especially in acute situations when the symptoms occurred. Actively working on their mental health, even between therapy sessions, helped some of the students to better cope with symptoms, which was often associated with greater control over the disease, increased self-initiative, and self-efficacy:

The app gave me the opportunity to distract myself and do something in the time between sessions when I felt bad or when I couldn’t get things done. At least I felt like I was actively doing something about my condition and trying to change it somehow. That really helped me.

P3

In addition, some participants reported that they had become much more aware of their strengths, which made them feel that they were not at the mercy of their disease. Taking time to work with the app seemed to help them reflect on and learn more about themselves:

Well, I’ve realized that I actually have a lot of resources and that I’m not so helpless. I can take initiative myself and I’ve noticed how that’s helped me.

P28

#### Psychoeducation

Almost all participants mentioned that they increased their knowledge about depression or anxiety disorders, which included the awareness of specific symptoms as well as of biopsychosocial factors that might influence the progression of their disease. In addition, some participants emphasized that the knowledge gain was helpful in counteracting emerging symptoms. In particular, for those who did not have therapy experience yet or had little disease-specific knowledge, the app seemed to serve as a good tool for psychoeducation:

I’ve never done any therapy or anything like that before, and I think it was really good to improve your psychoeducation, just to gain some knowledge about the subject.

P38

Furthermore, learning about the mental disorder seemed to facilitate its acceptance as a disease that can be treated and to decrease the fear of not receiving adequate help:

It really helped me realize what was going on with me because you often criticize yourself. I also did that because I had no other explanation for my behavior...You can cope with it better if you have explanations, and that was really helpful for my environment and me.

P80

Moreover, some students mentioned that the content of the app modules made them feel that they were not the only ones dealing with depression or anxiety:

So, you’re not alone. There are other people dealing with the same things. And it can actually be treated somehow. Because that was something I was really scared of at the beginning; I was afraid that it wouldn’t get better...And that’s what I liked best about the modules.

P20

As a suggestion for improvement, some participants wished for more personalization options, which would allow users to skip specific exercises or time-consuming tasks that were perceived as unimportant for their personal situation. In addition to the psychoeducational chapters, one participant proposed the integration of a take-home message into the app, which might help students in their daily lives when the bCBT was over:

Well, I’ve really learned a lot about the disease so far. What I missed somehow was some kind of instruction at the beginning, like...you can do this and that now, when the final sessions was over, or this is how you can continue on your own. You weren’t told that right away and, well, this might still be kind of nice.

P48

### Facilitators of the Use of the App

#### Usability and Structure

Most of the interviewed participants agreed that the app was well structured and easy to navigate, irrespective of the included onboarding function and without prior technical knowledge. The standardized structure of the tasks, which always included the theoretical background of a specific topic, a general example, and the possibility of directly applying it to oneself, was highlighted as helpful. In addition, the intuitive handling as well as the appropriate, well-chosen language were mentioned as facilitators of app use:

I’m not sure if an onboarding is really necessary, because the app is simply well-structured. You can immediately find the headings of the different subcategories, for example. Everything is designed to be very simple.

P66

Some participants explained that they would have benefited from an integrated therapy plan as an orientation to their therapy process. To find their way around the app more quickly, the interviewed students also indicated that it would have been advantageous to immediately see which exercises had already been completed and which were still to come:

I think I would have liked to have an overview at the beginning, some kind of therapy plan that is displayed in the app as a schedule. So that you exactly know at which point in therapy you are right now, what is still ahead of you, what is more to come, to have a rough orientation.

P15

Furthermore, receiving praise after completing a task seemed to encourage some participants to continue and was perceived as a confirmation of their progress. However, a few participants criticized the lack of direct, detailed feedback on completed tasks, which could be integrated into the app and might help them develop further:

You just answer the questions in the app, but you’re kind of stuck with them and you don’t necessarily get on. So, you don’t necessarily get feedback that directly helps you.

P46

In addition, some participants proposed including additional notifications in the app that would remind users to complete tasks before the next therapy session. In their opinion, this could have increased overall therapy adherence:

It would be nice to be reminded every now and then that you still have to do the tasks...I mean, you shouldn’t get notifications all the time, I think that would be annoying, but a reminder just before the session might be nice. You should get a notification if you haven’t done anything yet. I think that would be convenient for me.

P64

#### Content and Design

Although some participants would have liked more colors in the app, most interviewed students agreed that the simple design was friendly and inviting, which facilitated the use and reduced overload. According to them, the clear structure invited users to try out many different tasks and topics offered by the app:

It has a very appealing design, but it’s still neutral. I really liked that a lot. And I really liked that there is a personal form of address, even with my name. And it’s nice that you can select everything, that there are the courses, the journal or the resource kit, for example. That you don’t have everything at once, but that everything is structured well and split in smaller parts.

P49

The multimedia components of the app, such as videos or audio contents, were considered to be professionally produced. They were regarded as neither too long nor too short but just right for daily use by most participants. In addition, some students appreciated the personal component of seeing people explain different things in the videos. Overall, most participants stated that the content was well prepared and presented:

Well, the videos or the features where you could hear a voice, that was something that made the app really individual...Sometimes, the videos were just a couple of seconds long. Something was shortly explained, but it was really helpful for me to actually see or hear someone.

P8

#### Support

Many participants valued the app as a caring companion or “guardian angel” (P49) because they felt that they were not left alone with their problems between therapy sessions and were able to stay on track. The app served as an anchor, providing stability, especially when there was no one else to talk to:

Especially, when I wasn’t doing well and there were no other people I could have talked to, well, for example when I was up really late because of the depression, then it’s harder to reach out to a friend or someone, and it was nice to have some kind of support or anchor if you needed it. And also, to have some guidance, something that gives you hope and the tools you need.

P39

Whereas many participants seemed to have built a strong, personal connection to the app, others simply regarded it as a helpful tool in addition to face-to-face therapy sessions:

I think the app is just an app. I use it as a tool to work with someone towards a particular goal, and that’s it.

P4

Moreover, the app seemed to provide a structure for the therapy process for some users, and this was perceived as beneficial for the overall experience:

I can definitely say that it made me feel more comfortable in therapy. It made me realize: Alright, there’s a certain structure and I know what to expect. I’ve done therapy before, about ten years ago, and some of the things there were really annoying: I was like, okay, every time I come here, we’re doing the same thing, and if there’s homework, it is not even discussed. It was so annoying to have no structure at all. And here, I knew that the therapist was going to ask me if I did my exercises. I really liked that.

P45

### Barriers to the Use of the App

#### Overwhelming Emotions

Apart from the perceived advantages of the app, some students expressed concern about being left alone with feelings and emotions that might arise when dealing with the tasks and reflections. A few participants mentioned that this could be problematic, especially in acute situations, which would make it even more important to carefully assign tasks to different users:

In very acute cases, when it would be easiest for the person to deal with their emotions with someone else. Depending on the depth of the tasks, it might be critical if the person has to manage it alone. But I think therapists also choose tasks for their patients, if I understood correctly. And that’s something that they could then pay attention to.

P6

Furthermore, some participants indicated that being asked to complete the exercises until the next therapy session might lead to more pressure and overload for those who already had difficulties in managing daily tasks. They noted that, in particular, students with depressive symptoms might find it taxing to work with the app in addition to face-to-face sessions. As they often had very limited energy levels and sometimes already struggle with daily tasks, they might perceive an additional digital component as an extra workload and find it overwhelming:

Well, I can imagine that it can lead to an overload, especially in cases of severe depression, when you’re struggling with lethargy anyway, and then you feel obliged to do these tasks.

P3

#### Time Requirement

In accordance, a few participants noted that the increased total time spent with oneself might be a burden. From their point of view, the feeling of not being able to successfully complete the assigned tasks, for example, because of time constraints, could further increase users’ self-doubt:

Because you always think: Alright, I have to remember this, or I still have to do that. And then I think I would feel like having failed, because I didn’t get it done. And I would be really dissatisfied with it and also with myself, which is not really beneficial.

P59

Another individual barrier to using the app might be the increased engagement with the smartphone, which could also be perceived as a stressor for those who wanted to be less digitally involved:

I think, and I’ve noticed this with myself, if you’re in a relatively stressful phase and you actually want to get away from your smartphone and use fewer digital devices, and at the same time you know that you’re dependent on using this app, then it’s definitely a stressor. But I think that’s very individual.

P15

#### Data Security Concerns

Overall, participants did not seem to have any concerns about data security and were not worried about submitting data related to their health status because the app provided detailed explanations about how the data would be handled to maintain anonymity and confidentiality:

I didn’t have any concerns. You were always told that data were not given to anyone else, also after having finished a task. I really liked always getting an overview over my data.

P10

## Discussion

### Principal Findings

This study evaluated the potential benefits of a bCBT program accompanied by *elona therapy* as well as the facilitators of and barriers to its use among university students with mild to moderate depression or anxiety symptoms. The 6-week bCBT intervention comprised 6 face-to-face individual CBT sessions via videoconferencing combined with the depression or anxiety module of the app. Although there are some qualitative studies covering health care providers’ [40] and patients’ experiences with bCBT [41] as well as with stand-alone iCBT [54], this study is, to our knowledge, the first to investigate factors related to the use (and nonuse) of an app within a bCBT program among students.

Predominantly, students reported positive experiences with the app and listed disorder-specific knowledge gain, the transfer of the therapy content into daily life, and impulses for self-reflection as general benefits. In accordance with the results achieved by Wu et al [30], who conducted a quantitative study with patients with symptoms of anxiety or depression, our results show that using DTx in bCBT can be especially useful for psychoeducation, as it helps to reinforce the uptake of the content of therapy sessions, such as coping skills, and key concepts such as the biopsychosocial framework [55]. This result seems to be particularly important, as research has shown that students lacking coping responses may be at risk for psychopathology when faced with high levels of stress, for example, stress related to the COVID-19 pandemic [56]. In addition to prior research showing that bCBT is effective in reducing symptomology [29-32], the results of our qualitative investigation further indicate that the use of the app along with face-to-face sessions also seems to be associated with positive feelings regarding the therapy process among students. Specifically, our results indicate that many students gained the impression that their therapy would have been less structured and less effective without the app, which was also mentioned by participants in the study by Urech et al [42]. Furthermore, the app was considered beneficial in complementing face-to-face sessions because it initiated the active management of symptoms and encouraged self-reflection. Many participants evaluated the app as a supportive tool and caring companion, which has been echoed by prior research on the ability of DTx to potentially prevent a therapeutic drift between face-to-face therapy sessions [42,45] but not yet for the specific target group of students. This implies that there might have been a clear concept of how the app was embedded in the overall therapy program. However, to increase the acceptance and dissemination of such treatment modes in low-resource settings, such as student counseling centers, further real-world research needs to focus on how bCBT programs could be incorporated to reach students in need of psychological support. Our study lays the important foundation that it may be worthwhile to embed bCBT programs in the university context because they are perceived as helpful treatment options.

Similar to the findings by Urech et al [42], the implementation of the therapy content and behavioral modifications into daily life were mentioned as facilitators of the use because this seemed to help students initiate helpful and effective changes in their lives. Some interviewed students highlighted the advantage of being able to work independently with the app at their own pace, anytime and anywhere, which seemed to match students’ needs for location- and time-independent psychological support. The relatively short exercises that could be easily integrated even during stressful periods seemed to be essential for the uptake of the app. This result supports the findings of Stawarz et al [57], who claimed that the technology used in bCBT needs to provide simple, easy-to-understand content to prevent potential barriers owing to, for example, the lack of drive. No technical issues were mentioned as hindering factors. This seems important because it has been shown that technical problems result in frustration and anger, which can distract the therapy process [58,59].

Regarding the structure of the app, the comprehensive language and the clear design were mentioned as facilitators of the app use. A study by Fleischmann et al [60] showed that students might indeed feel overwhelmed by a cluttered design. Most students highlighted the intuitive handling, which made it possible to navigate through the app without prior technological knowledge. In contrast to the findings of Jakob et al [39], who found that skipping the tutorial significantly increased the chance of dropping out, the onboarding function of the app was evaluated as positive but not essential for the therapy process. This also appears to be a very important finding for health care providers because the fear that some students might have too little experience with new technologies and might not be familiar with the respective tools has been shown to be a barrier to the prescription of DTx [61,62]. In comparison to previous findings, data security concerns were rarely mentioned as potential barriers to app use [63]. This could be explained by the detailed descriptions of how data privacy was protected that were offered in the app as well as the students being digitally native. In line with the suggestions of Jakob et al [39] regarding how DTx should be designed to increase adherence, these explanations might have potentially reduced data privacy concerns.

To combine the potentials of both treatment modalities, DTx and face-to-face therapy sessions, a few participants mentioned that the app applied in this study could have benefited from even more personalization and interaction options that are optimally targeted at the students’ abilities, needs, and preferences [17]. Previous research has shown that personalization options that would, for example, allow users to skip specific exercises or time-consuming tasks, are perceived as crucial for the engagement with DTx among students [37,39], and this feature was not provided in this bCBT program. According to Stawarz et al [57], the ability to monitor the therapy progress could have potentially increased user engagement and therapy adherence, which was also mentioned by some participants. Similarly, allowing the therapist to keep track of the student’s process could have helped reinforce the connection to face-to-face sessions even more [45]. To further increase the adherence, some students suggested including additional reminders in the app, and this is in line with the conclusions of the systematic review by Jakob et al [39], who mentioned that reminders in the form of customizable push notifications can increase user engagement. However, this suggestion needs to be regarded with caution, as reminders can also be perceived as possible stressors for users [45,60]. Finally, some students raised concerns that patients with anxiety or depressive symptoms might feel overwhelmed by using the app, as working with the content might force them to address their problems without direct supervision. This implies that even in a bCBT, which includes face-to-face therapy sessions, there is a need to focus on emergency options when designing future DTx for students. Accordingly, Urech et al [42] showed that there seem to be different advantages and disadvantages of bCBT depending on the severity of patients’ depressive symptoms. Although patients with mild to moderate depressive symptoms mentioned no disadvantages, patients with moderately severe to severe symptoms noted the lack of an additional contact possibility through the web-based tool as well as the necessity of preexisting computer skills as possible barriers. As more pronounced depressive symptoms have been shown to be associated with lower adherence to self-management apps [39], the inclusion of personal contact options in case of emergencies and frequent guidance, especially for patients with severe symptoms, seems to be essential for effective bCBT programs. In addition, Arean et al [64] suggested including tasks that are specifically designed to target certain cognitive deficits implicit in depressive disorders, such as cognitive control, to increase adherence among patients with more severe symptoms. As we only included patients with mild to moderate depression symptoms, future research could clarify whether bCBT programs are also suitable for student groups with more severe symptoms.

Interestingly, our study showed that the perceived benefits of this bCBT program are in line with many of the explored expectations of patients receiving standard CBT but who have not yet had an experience with blended formats [38,49]. This study confirms that inexperienced bCBT users’ needs for personalization, integration into daily life, psychoeducation, and self-reflection options match the experiences of participants in this bCBT program. However, participants also called for more interactive therapeutic activities, which patients inexperienced in bCBT did not mention as a preference [38].

As a next step, it will be interesting to determine how patients, including depressed students, respond to the presented bCBT program *elona therapy depression* under real-world conditions. Since December 2022, the described bCBT program *elona therapy depression* has been listed as a digital health app (DiGA in German, so-called “app on prescription”) in the DiGA directory of the German Federal Institute for Drugs and Medical Devices. This means that since then, physicians and psychotherapists can prescribe this program for patients with depression at the expense of statutory health insurance companies.

### Strengths and Limitations

The strengths of this study include the large sample size of 102 interviewed students who did not receive any financial compensation, neither for their participation in the bCBT program nor for the interview, which might be associated with a high willingness to participate and a strong need to receive quick, low-threshold support for their mental health. Moreover, our study provides the first qualitative insights into the facilitators and barriers that might influence the uptake of an app in a bCBT program among students with mild to moderate depression or anxiety symptoms. Furthermore, almost all participants completed the bCBT program; hence, the therapy adherence seems to be substantially higher than that in study designs using either only face-to-face therapy sessions or DTx alone [41,65]. This leads to the assumption that the combination of individual face-to-face therapy sessions with personalized digital components could contribute to students’ motivation and engagement. Further research could investigate this hypothesis in a larger randomized controlled trial design, allowing for the comparison between bCBT and face-to-face CBT.

Our study has several limitations that need to be considered. As our data are qualitative in nature, the results are partially context specific to the *elona therapy* app for treating depression and anxiety, which was used in this study in students in Germany, and only potentially generalizable to universal bCBT topics or different contexts. Owing to the novelty of this bCBT program and the app, the focus of the interviews was predominantly on the app itself and less so on the face-to-face aspect. Future research could investigate how to optimally design and integrate face-to-face sessions along with the app use. It might be possible that a selection bias in the recruiting process occurred and that only students who were already willing and motivated to start therapy were recruited. In addition, students who participated in our study might have had a more positive attitude toward web-based therapy than nonresponders. Furthermore, we could only interview participants who had completed the 6-week bCBT program and not the dropouts (n=5). Therefore, it was to be expected that the opinions regarding the use of the digital health app and overall experiences with the bCBT program would be relatively positive. Moreover, most of the interview participants were female (89/102, 87.2%). Hence, our results might overrepresent the experiences of female participants in bCBT programs. In addition, we did not differentiate between students with depressive symptoms and students with anxiety disorders in the analysis of the data, although we interviewed almost twice as many students with depressive symptoms. Thus, we cannot draw any conclusions regarding whether bCBT might be more convenient for either one of the 2 groups or whether different patient groups have different needs. In addition, as the goal was to recruit only students with mild to moderate symptoms of depression or anxiety, there were no students with severe symptoms. Hence, we cannot conclude whether this target group would have reported similar experiences with the bCBT program applied in this study. Finally, face-to-face therapy sessions lasted only 25 minutes, which is shorter than typical therapy sessions and in line with the report of some of the participants who would have wished for longer sessions.

### Conclusions

Our study qualitatively investigated the potential benefits and limitations of a bCBT program accompanied by the *elona therapy* app as well as the facilitating and hindering factors to its use for symptoms of depression or anxiety among students. We were able to systematically classify the benefits, barriers, and facilitators using inductively developed themes and categories. We discussed our findings in terms of their similar and different implications for existing research in this field. These findings can be beneficial for researchers and developers of new DTx. To increase the dissemination of bCBT programs among students, further research could focus on how such treatment options could be incorporated into the university setting.

## Appendix

Multimedia Appendix 1 COREQ (Consolidated Criteria for Reporting Qualitative Research) checklist.

## Abbreviations

bCBT: blended cognitive behavioral therapy
CBT: cognitive behavioral therapy
COREQ: Consolidated Criteria for Reporting Qualitative Research
DTx: digital therapeutics
iCBT: internet-based cognitive behavioral therapy

## Data Availability

The qualitative data sets generated and analyzed in this study are not made available because publication could limit the protection of the informants, and such publication was not covered by the informed consent to participate in the study.
